# Clinical significance of dual-time-point 18F-FDG PET imaging in resectable non-small cell lung cancer

**DOI:** 10.1007/s12149-015-1013-3

**Published:** 2015-08-08

**Authors:** Katsuhiko Shimizu, Riki Okita, Shinsuke Saisho, Takuro Yukawa, Ai Maeda, Yuji Nojima, Masao Nakata

**Affiliations:** Department of General Thoracic Surgery, Kawasaki Medical School, 577 Matsushima, Kurashiki, Okayama 701-0192 Japan

**Keywords:** Non-small cell lung cancer, Lymph node metastasis, Dual-time-point imaging 18F-FDG PET, Retention index

## Abstract

**Objective:**

The maximal standardized uptake value (SUVmax) of pulmonary lesions on dual-time-point (DTP) fluorodeoxyglucose positron emission tomography (FDG-PET) has been shown to be useful for differentiation between malignant and non-malignant pulmonary lesions, and also to be of value for intrathoracic nodal staging of non-small cell lung cancer (NSCLC). However, a few NSCLC lesions have been found to show decreased FDG uptake on delayed images, and the significance of this finding remains unknown.

**Patients and methods:**

We conducted a retrospective review of the data of 284 patients with NSCLC who underwent DTP FDG-PET before surgery. Cases of adenocarcinoma in situ and minimally invasive adenocarcinoma were excluded, because these lesions show little FDG uptake. Each patient was scanned at 60 min (early acquisition; SUV-E) and 115 min (delayed acquisition; SUV-D) after the radiopharmaceutical injection. The intratumoral retention index (RI) of 18F-FDG was measured for each examination by the DTP method. Recurrence-free survival (RFS) was determined by the Kaplan–Meier method and compared in relation to the SUV-E, SUV-D, and RI by univariate and multivariate analysis using models including the clinico-pathological prognostic factors.

**Results:**

Of the 284 cases, the RI ≤ 0 was in 49 cases (17.3 %). This group of patients showed lower values of SUV-E and SUV-D, a smaller tumor size, and a lower rate of lymphatic invasion or vascular invasion. It was particularly noteworthy that lymph node metastasis was not histopathologically confirmed in any of these patients. Univariate analysis identified the RI, SUV-E and SUV-D, besides age, tumor size, lymph node metastasis, and tumor differentiation grade as predictors of the RFS. On the other hand, multivariate analysis identified the RI and lymph node metastasis, but not the SUV-E and SUV-D, as independent predictors of the RFS.

**Conclusions:**

This study demonstrated that DTP FDG-PET of the primary tumor in NSCLC can be useful to predict the RFS of the patients. In addition, this method may also be useful to predict the presence/absence of intrathoracic lymph node metastasis in these patients.

## Introduction

Fluorodeoxyglucose positron emission tomography (FDG-PET) has become an important tool for the diagnosis and staging of non-small cell lung cancer (NSCLC) [1]. The maximal standardized uptake values (SUVmax) on FDG-PET is calculated as the ratio of the activity in the tissue per unit volume to the injected dose by body weight, and is widely used because of the simplicity of its measurement [2]. In NSCLC patients, the SUVmax values of the primary tumors have been known to be correlated with the disease stage, nodal status, histological type, tumor differentiation grade, and rate of progression of the tumors [3–5]. In addition, high SUVmax values have been reported as being among the poor prognostic factors in patients with NSCLC [5–7].

Several studies have reported that determination of the SUVmax of pulmonary lesions on dual-time-point (DTP) FDG-PET is useful for differentiation between malignant and non-malignant pulmonary lesions [8–10]. Moreover, several investigators have shown that determination of the SUVmax of the lymph nodes on DTP FDG-PET improved the diagnostic ability of this imaging modality for intrathoracic nodal staging in NSCLC patients [11–13]. In general, most malignant lesions, including primary tumors and lymph nodes, also show increased FDG uptake on the delayed-phase images.

However, it has been reported that some NSCLC lesions actually show decreased FDG uptake on the delayed-phase images [14]. The significance of such decreased FDG uptake of NSCLC lesions remains unknown. In the present study, we analyzed the diagnostic impact of DTP FDG-PET for intrathoracic nodal staging and also its prognostic impact in patients with resectable NSCLC.

## Patients and methods

### Study population

We conducted this retrospective study in a total of 284 patients, who had undergone DTP FDG-PET before surgery, which consisted of surgical resection with lymph node dissection or sampling, at the Kawasaki Medical School Hospital between 2007 and 2013. None of the patients had received either radiotherapy or chemotherapy prior to the surgery. The TNM stage was determined according to the revised criteria published in 2009. The histological diagnosis of the tumors was based on the criteria of the IASLC/ATS/ERS proposed in 2011 [15]. Cases of adenocarcinoma in situ and minimally invasive adenocarcinoma were excluded, because these lesions show little uptake of FDG [16]. This study was conducted with the approval of the institutional Ethics Committee of Kawasaki Medical School (No.1323). Follow-up information until either recurrence or December 2014 was obtained from the medical records.

### Fdg-pet

All PET/CT examinations were performed with a dedicated PET/CT scanner (Discovery ST Elite; GE Healthcare, Japan). The axes of the multidetector CT and PET systems were mechanically aligned so that the patient could be moved from the CT to the PET scanner gantry by simply changing the position of the examination table. The resultant PET and CT scans were coregistered with hardware. PET/CT scanning was performed at 60 and 115 min after the intravenous injection of 150–220 MBq of ^18^FDG (FDGscan, Universal Giken, Nihon Mediphysics, Tokyo, Japan). The regions of interest (ROI) were placed three-dimensionally over the lung cancer nodules. Semi-quantitative analysis of the images was performed by measuring the SUVmax of the lesions. The SUVs were calculated using the following equation: tumor activity concentration/(injected dose/body weight). Therefore, the SUVmax measurements of the primary tumor were obtained both on early- (SUV-E) and delayed scans (SUV-D). In this study, we analyzed the “no integration of FDG” as “zero”. In addition, all the cases with SUV-E value of zero also had SUV-D value of zero. Furthermore, we calculated the retention index (RI) from the SUVmax values by the following formula: RI (%) = (SUV-D−SUV-E)/SUV-E × 100 when SUV-E > 0, and RI (%) = 0 when SUV-E = 0. RI is really impossible calculation with SUV-E and SUV-D value of zero, therefore, we considered the value as zero.

### Statistical analysis

Statistical analysis was performed for examining the significances of differences and the possible correlations between the RI on DTP FDG-PET, and the clinicopathological features; Fisher’s exact test or the *χ*^2^ test was used, as appropriate. An unpaired *t* test was used for comparison of continuous data. The prognostic evaluation was based on the recurrence-free survival (RFS). RFS was defined as the time from the date of surgery until lung cancer recurrence or non–lung cancer death. The survival curves were estimated using the Kaplan–Meier method, and differences among the curves were evaluated by the log-rank test. Univariate and multivariate analysis were performed using Cox proportional hazard models. All the statistical analyses were conducted using the SPSS software (Version 17.0; SPSS Incorporation, Chicago, IL). All statistical tests were two-sided, and probability values of <0.05 were regarded as denoting statistical significance.

## Results

### Clinical characteristics

The characteristics of the patients are summarized in Table 1. The patients ranged in age from 37 to 83 years (mean, 70.4), and there were 195 men and 89 women. The majority of patients (184, 64.8 %) had adenocarcinoma, while 71 (25.0 %) had squamous cell carcinoma, 16 (5.6 %) had large cell carcinoma, and 13 (4.6 %) had other histological types. Pathological N0 disease was confirmed in 230 patients (81.0 %), and N1 or N2 disease in 54 patients (19.0 %). The median follow-up period was 33.9 months (range 5–72 months).

**Table 1:** Patient characteristics (*n* = 284)

Variable	Number	%
Age, mean ± SD	70.4 ± 8.9	
Sex		
Male	195	68.6
Female	89	31.4
Histology		
Adenocarcinoma	184	64.8
Squamous cell	71	25.0
Large cell	16	5.6
Others	13	4.6
Pathological lymph node status		
N0	230	81.0
N1	22	7.7
N2	32	11.3
Pathological stage		
IA	120	42.2
IB	82	28.9
II(A + B)	42	14.8
III(A + B)	40	14.1
Surgical procedure		
Pneumonectomy	2	0.7
Lobectomy	211	74.3
Segmentectomy	30	10.6
Wedge resection	41	14.4

### Pathological characteristics in relation to the SUV-E, SUV-D, and the RI

Table 2 shows the SUV-E, SUV-D and RI values in relation to the pathological findings. The mean SUV-E was 7.3 (range 0–30.7) and the mean SUV-D was 8.9 (range 0–35.7). In relation to the histological type, the SUV-E and -D values were significantly higher in the non-adenocarcinoma group than in the adenocarcinoma group. The SUV-E and -D values were significantly higher in cases with a large tumor sizes (>31 mm), moderate or poor tumor differentiation grade, and pathological lymph node metastasis, lymphatic invasion, or vascular invasion, than in those with small tumor sizes (<30 mm), well-differentiated tumors, and no pathological lymph node metastasis, lymphatic invasion, or vascular invasion. The mean RI was 21.1 % (range −23.1 to 214.0 %). The RI values were significantly higher in the cases with non-adenocarcinomas, moderate or poor tumor differentiation grade, and pathological lymph node metastasis, or vascular invasion than in those with adenocarcinoma, well-differentiated tumors, and no pathological lymph node metastasis or vascular invasion.

**Table 2:** Pathological characteristics in relation to the SUV-E, SUV-D, and the RI

Characteristics	SUV-E	SUV-D	Retention index (%)
All cases	7.3 ± 5.8	8.9 ± 7.0	21.1 ± 21.6
Histology			
Adenocarcinoma	5.4 ± 5.0	6.5 ± 6.1	18.4 ± 18.8
Non-adenocarcinoma	10.9 ± 5.4	13.4 ± 6.3	26.0 ± 25.4
*p* value	<0.001	<0.001	0.010
Tumor differentiation			
Well	3.3 ± 3.3	4.0 ± 4.1	15.3 ± 19.0
Moderate/poor	9.2 ± 5.7	11.3 ± 6.9	23.9 ± 22.2
*p* value	<0.001	<0.001	0.001
Tumor size			
<30 mm	5.0 ± 4.1	6.2 ± 5.2	22.0 ± 25.4
>31 mm	10.8 ± 6.2	12.9 ± 7.4	19.7 ± 14.2
*p* value	<0.001	<0.001	0.337
Pathological nodal status			
Negative	6.6 ± 5.6	8.0 ± 6.9	19.7 ± 19.1
Positive	10.5 ± 5.3	13.0 ± 6.2	27.1 ± 29.4
*p* value	<0.001	<0.001	0.024
Lymphatic invasion			
Negative	6.6 ± 6.0	8.0 ± 7.2	20.0 ± 23.2
Positive	9.6 ± 4.6	11.8 ± 5.5	24.5 ± 15.1
*p* value	<0.001	<0.001	0.065
Vascular invasion			
Negative	4.8 ± 4.8	5.9 ± 6.0	18.9 ± 25.5
Positive	10.4 ± 5.4	12.7 ± 6.3	23.8 ± 15.1
*p* value	<0.001	<0.001	0.045

### Clinicopathological characteristics in relation to the RI

**Fig. 1:**
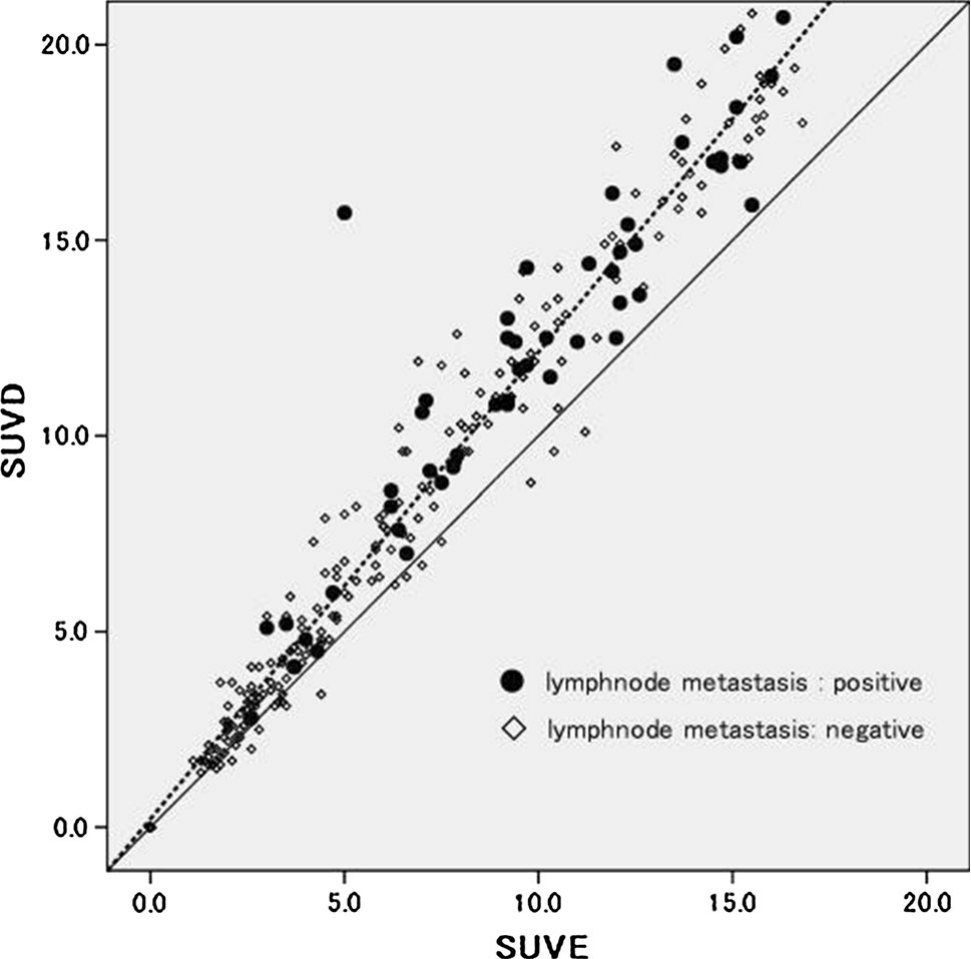
A scattergram showed that a significant positive correlation was observed between the SUV-E and SUV-D (*r* = 0.971, *P* < 0.001), and none of the all cases with RI ≤ 0 had lymph node metastasis

We categorized the 284 patients according to the RI: RI ≤ 0 (Group A; *n* = 49: 17.3 %) or RI > 0 (Group B; *n* = 235). Group A had lower SUV-E (*p* < 0.001) and SUV-D (*p* < 0.001) values, a smaller tumor size (*p* = 0.001), well differentiation tumor grade (*p* = 0.001), and a smaller percentages of patients with lymph node metastasis (*p* = 0.001), pleural invasion (*p* < 0.001), lymphatic invasion (*p* < 0.001), and vascular invasion (*p* < 0.001) than Group B, while no significant association was observed with the age (*p* = 0.381), or sex (*p* = 0.339) (Table 3). It was particularly noteworthy that lymph node metastasis was not confirmed histopathologically in any of the patients with RI ≤ 0. A scattergram showed the SUV-E and SUV-D values in relation to the presence/absence of lymph node metastasis (Fig. 1). A significant positive correlation was observed between the SUV-E and SUV-D (*r* = 0.971, *P* < 0.001), and none of the all cases with RI ≤ 0 had lymph node metastasis irrespective of the SUV-E or SUV-D value.

**Table 3:** Clinico-pathological characteristics in relation to the RI

Characteristics	*N*	RI ≤ 0	RI > 0	*P* value
Patients (number)	284	49	235	
SUV-E (mean)		2.2	8.4	<0.001
SUV-D (mean)		2.0	10.4	<0.001
Age (mean), year		69.5	70.6	0.381
Sex				
Male	195	31	164	0.399
Female	89	18	71	
Histology				<0.001
Adenocarcinoma	184	44	140	
Non-adenocarcinoma	100	5	95	
Tumor size (mean), mm		22.5	30.5	0.001
Pathological nodal status				<0.001
Negative	230	49	181	
Positive	54	0	54	
Tumor differentiation				<0.001
Well	92	34	58	
Moderate	110	12	98	
Poor	82	3	79	
Pleural invasion				<0.001
Negative	185	44	141	
Positive	99	5	94	
Lymphatic invasion				<0.001
Negative	214	47	167	
Positive	70	2	68	
Vascular invasion				
Negative	157	44	113	<0.001
Positive	127	5	122

### Histological Characteristics of the cases showing RI ≤ 0 on DTP FDG-PET

Table 4 shows the SUV-E, SUV-D, and RI values in relation to the histological subtype of NSCLC in patients with RI ≤ 0. The most frequent subtype was lepidic-predominant invasive adenocarcinoma (*n* = 24), followed in frequency by papillary-predominant invasive adenocarcinoma (*n* = 8), acinar-predominant invasive adenocarcinoma (*n* = 6), invasive mucinous adenocarcinoma (*n* = 6), and squamous cell carcinoma (*n* = 5). The SUV-E and SUV-D values of acinar-predominant invasive adenocarcinoma and squamous cell carcinoma were higher than those of lepidic-predominant invasive adenocarcinoma. The RI of squamous cell carcinoma was higher than that of lepidic- predominant invasive adenocarcinoma.

**Table 4:** Histological characteristics of the cases showing RI ≤ 0 on DTP FDG-PET (*n* = 49)

Characteristics	*n*	SUV-E	SUV-D	Retention index (%)
Lepidic-predominant invasive AD	24	0.98	0.93	−2.1
Papillary predominant invasive AD	8	2.25	2.01	−5.5
Acinar-predominant invasive AD	6	3.00*	2.85*	−3.1
Invasive mucinous AD	6	1.83	1.78	−1.8
Squamous cell carcinoma	5	7.20*	6.58*	−8.7*

*AD* adenocarcinoma

* SUV-E: Lepidic vs. Acinar, Lepidic vs. Squamous: *P* < 0.05

* SUV-D: Lepidic vs. Acinar, Lepidic vs. Squamous: *P* < 0.05

* RI: Lepidic vs. Squamous: *P* < 0.05

### Analysis of the prognosis

Univariate analysis identified the age, tumor size, nodal metastasis, tumor differentiation grade, SUV-E, SUV-D, and RI as predictors of the RFS. On the other hand, multivariate analysis using a Cox proportional hazard model identified only age (*P* < 0.001) and nodal metastasis (*P* < 0.001), and the RI (*P* = 0.023) as independent predictors of the RFS (Table 5).

**Table 5:** Analysis of the prognosis

	Univariate			Multivariate		
	HR	95 %CI	*p* value	HR	95 %CI	*p* value
Sex						
Male/female	1.36	0.84–2.19	0.209			
Age						
≥70/<70	2.43	1.54–3.85	<0.001	2.45	1.54–3.92	<0.001
Histology						
AD/non AD	0.66	0.43–1.02	0.064			
Pathological *T*						
≥30 mm/<30 mm	2.11	1.37–3.24	0.001	1.27	0.77– 2.01	0.345
Pathological *N* factor						
Positive/negative	5.78	3.75–8.91	<0.001	4.63	2.92–7.36	<0.001
Tumor differentiation						
Mod + por/well	2.64	1.53–4.55	0.001	1.49	0.82–2.72	0.194
SUV-E	1.08	1.04–1.11	<0.001	1.20	0.96–1.51	0.106
SUV-D	1.06	1.03–1.09	<0.001	0.87	0.72–1.04	0.117
RI-SUVmax						
RI > 0/RI ≤ 0	6.93	2.19–21.95	0.001	4.03	1.19–13.63	0.025

## Discussion

This study demonstrated that DTP FDG-PET imaging of the primary tumors can be useful to predict the RFS in NSCLC patients. In addition, this method may also be useful to predict the presence of intrathoracic lymph node metastasis. It was particularly noteworthy that lymph node metastasis was not confirmed histopathologically in any of the patients with RI ≤ 0 on DTP FDG-PET. In addition, we demonstrated that all the cases with SUV-E value of zero also had SUV-D value of zero. Therefore, delayed scan can be omitted when the early scan does not show FDG accumulation in the tumor. This is the first report of investigation of the significance of decreased FDG uptake in the pulmonary lesions of NSCLC.

The SUV measured on FDG-PET is a semi-quantitative measure of the degree of glucose uptake in a lesion. Determination of the SUVmax of primary lung nodules has been reported to be helpful for distinguishing between malignant and benign tumors, based on the relatively higher values in malignant tumors [17]. In 2001, Kubota et al. reported that most malignant lesions, including primary lung cancers, mediastinal node metastases, and lymphomas, showed higher FDG uptake at 2 h than at 1 h after injection of FDG, while normal tissues and benign lesions, except for sarcoidosis, showed a lower uptake at 2 h than at 1 h after injection; they proposed that, therefore, FDG-PET scans obtained in the delayed-phase show a better sensitivity than the early-phase images [18]. The SUVmax has been established as a powerful predictor of the treatment outcome in NSCLC patients. However, many factors, such as the patient preparation method, procedure, scan acquisition parameters, image reconstruction method, and data analysis method employed, are known to affect the SUVmax [19, 20]. For example, Westerterp et al. described that the reported variations of up to 30 % in SUV values from 3 institutions could pose a serious problem when performing multicenter studies [21]. In fact, differing values of the SUVmax for detecting lymph node metastasis have been reported from different studies. To overcome this disadvantage, we used the RI of the SUVmax using DTP FDG-PET. RI is a relative scale, and therefore does not require standardization. Based on these findings, we investigated the impact of RI on the prognosis in NSCLC patients.

Three steps are involved in the 18-F FDG accumulation in cancer cells: (1) facilitated diffusion through glucose transport proteins; (2) subsequent phosphorylation by hexokinase isoforms producing FDG-6-phosphate; (3) decreased dephosphorylation [22]. Thus, 18-F FDG accumulation depends basically on the rate of transport through the cell membrane and the activity of hexokinase. Theoretically, the ratio of hexokinase to FDG-6-phosphatase more specifically increases in tumors than in inflammatory lesions, resulting in a gradual accumulation of FDG and a further increase of the SUV on delayed imaging. Therefore, RI may be a better indicator of the malignant potential, whereas the SUV-E represents malignant potential of a tumor [23]. Thus, being a possibly better indicator of malignant potential, RI might be a predictor of the risk of nodal metastasis.

Evaluation of lymph node metastases is important for the staging of lung cancer and for the selection of the most appropriate therapy. Since FDG-PET has been shown to be superior to CT for evaluation of the lymph node status in patients with NSCLC, FDG-PET has been increasingly used worldwide [24, 25]. In general, the lymph node status is interpreted qualitatively or quantitatively on FDG-PET scans, based on the tendency towards a higher FDG uptake in metastatic lymph nodes than in non-metastatic lymph nodes [24, 25]. In addition, recently, several investigators have introduced DTP FDG-PET to improve the accuracy of FDG-PET for nodal staging in cases of NSCLC [11–13]. Based on the results of previous studies, it is known that malignant tumors exhibit increased FDG uptake for several hours, while benign lesions show decreased FDG uptake on DTP FDG-PET [9, 21]. While studies have generally shown favorable accuracy of this modality for nodal staging, this method is rather cumbersome for examining all of the lymph nodes showing FDG accumulation. In this study, we evaluated the primary tumors in NSCLC patients by DTP FDG-PET, which is a simpler and more accurate method than imaging of the number of lymph nodes involved.

To date, DTP FDG-PET has mainly been utilized to improve the accuracy of diagnosis of lung nodules or lymph nodes. Recently, few reports have addressed the possible usefulness of RI in predicting the risk of recurrence or prognosis. Kim et al. reported that RI proved inadequate for predicting the prognosis in terms of the OS and disease-free survival in early stage (stage I and II) NSCLC patients treated by surgical resection [26]. On the other hand, Satoh et al. reported that a higher value of the RI significantly predicted a higher risk of distant metastasis in stage I NSCLC patients treated by stereotactic body radiation. They suggested that the RI tended to predict lower risk of local recurrence and lymph node metastasis [23]. We also demonstrated the prognostic value of DTP FDG-PET for the RFS or OS in NSCLC patients.

This study has several limitations that should be considered when interpreting the results. The retrospective study design was a major limitation. Minor limitations included insufficient evidence of the validity of the 115 min of delayed acquisition and the cut-off value for RI.

## Conclusion

In conclusion, this study demonstrated that DTP FDG-PET imaging of the primary tumors in patients with NSCLC can be useful to predict the RFS in these patients. In addition, this method may also be useful to predict the presence/absence of intrathoracic lymph node metastasis.
